# Fear of COVID-19 and severity of particular autistic traits in the general population.

**DOI:** 10.1192/j.eurpsy.2023.370

**Published:** 2023-07-19

**Authors:** D. M. Bieczek, A. I. Ściślicka, A. V. Bobowska, F. M. Tomsia, K. M. Wilczyński, M. Janas-Kozik

**Affiliations:** ^1^Students’ scientific association at the Department of Psychiatry and Psychotherapy of Developmental Age; ^2^Department of Psychiatry and Psychotherapy of Developmental Age, Medical University of Silesia, Katowice, Poland

## Abstract

**Introduction:**

A lot of issues have raised since the beginning of the pandemic and doctors had to learn how to deal with increasing problems of stress and anxiety. The new situation was a great threat to one’s safety and some more vulnerable people could experience a higher level of anxiety. Patients with autistic traits might be more prone to it. It is essential to find out who is more exposed and who will require additional care.

**Objectives:**

The aim of this study was to assess the level of fear of COVID-19 and explore its possible correlation with the severity of the particular autistic traits in the general population.

**Methods:**

The study was conducted online, utilizing the questionnaire consisting of Autism-Spectrum Quotient (AQ) to assess the severity of autistic traits (social skills, attention switching, attention to detail, communication and imagination) and questionnaire FCV-19S that was used to assess the level of fear of COVID-19. Access to the questionnaire was possible from 16.02.2021 to 11.06.2021 and 214 unique records were gathered during this period.

**Results:**

In the multiple regression (R^2^= 0,16, p<0,0001) a positive relationship between the level of felt fear of COVID-19 and the severity of difficulties with attention switching (p=0,006) and age (p=0,000015) was found.

**Conclusions:**

People with higher severity of problems with attention switching demonstrated higher levels of fear of COVID-19 due to cognitive stiffness and disturbances in the regulation of emotions. Older people presented a higher level of fear as well.

**Disclosure of Interest:**

None Declared

